# Multifunctional PVA Hydrogels with Balanced Mechanical Properties, Passive Radiative Cooling, and Flame Retardancy via Synergistic Freeze–Thawing and Hofmeister Effect

**DOI:** 10.3390/polym18151869

**Published:** 2026-07-30

**Authors:** Kunkun Tu, Suhao Li, Jiayi Li, Jinjing Liu, Jianing Ji, Lining Dong, Jiaqi Li, Shuang Li, Zhongfei Liu, Ziyue He, Xinjian He, Huan Xu, Shihang Li

**Affiliations:** 1 Jiangsu Key Laboratory of Coal–Based Greenhouse Gas Control and Utilization, Carbon Neutrality Institute, China University of Mining and Technology, Xuzhou 221008, China; tuk@cumt.edu.cn (K.T.); jiayili@cumt.edu.cn (J.L.); liujinjing@cumt.edu.cn (J.L.); 2 School of Safety Engineering, China University of Mining and Technology, Xuzhou 221116, China; lisuhao2005@126.com (S.L.); 16244214@cumt.edu.cn (J.J.); 15194742@cumt.edu.cn (J.L.); 18628583990@163.com (Z.L.); xinjian.he@cumt.edu.cn (X.H.); 3 School of Environment Science and Spatial Informatics, China University of Mining and Technology, Xuzhou 221116, China; 05235713@cumt.edu.cn; 4 School of Materials Science and Physics, China University of Mining and Technology, Xuzhou 221116, China; 15863575101@163.com; 5 School of Electrical Engineering, China University of Mining and Technology, Xuzhou 221116, China; 23230410@cumt.edu.cn

**Keywords:** PVA hydrogel, Hofmeister effect, mechanical toughness, thermal management, passive radiative cooling, flame retardancy

## Abstract

Conventional poly(vinyl alcohol) (PVA) hydrogels struggle to integrate the mechanical robustness, thermal management, and fire safety demanded by extreme environments. To overcome this, we fabricate a multifunctional hydrogel via a synergistic strategy combining freeze–thawing and citrate-driven Hofmeister salting-out. Kosmotropic citrate ions aggressively strip polymer hydration shells, driving intense intermolecular hydrogen bonding, elevated crystallinity, and severe network densification. Consequently, the optimized cit@PVA hydrogel exhibits a balanced mechanical performance, achieving a tensile strength of 1.31 MPa and an elongation at break of approximately 150%. The citrate-induced network densification not only reinforces the mechanical integrity of the hydrogel but also regulates its thermal transport characteristics. Benefiting from the dense polymer framework and intrinsic infrared-active chemical structures, the cit@PVA hydrogel demonstrates excellent thermal management capability, including effective high-temperature thermal insulation and high mid-infrared emissivity (~85%) for passive radiative cooling. Furthermore, the incorporated citrate shifts the degradation pathway toward catalytic charring, rapidly forming a dense carbonaceous shield to completely prevent burn-through during direct flame exposure. This scalable structural design overcomes traditional performance limitations, creating resilient soft materials for advanced flexible electronics and smart protective wearables.

## 1. Introduction

Hydrogels are a class of materials with unique properties, including high water content, flexibility, and biocompatibility, making them ideal for a variety of applications in flexible electronics, soft robotics, and biomedical devices [1,2,3,4]. Poly(vinyl alcohol) (PVA) hydrogels have been widely studied due to their low cost, ease of synthesis, and environmental compatibility [5,6]. However, hydrogels are prone to structural collapse at high temperatures; flammable and may release toxic volatiles during combustion, and their limited mechanical strength and low environmental stability often restrict their use in load-bearing applications or systems requiring thermal regulation [7,8,9].

Among these critical drawbacks, overcoming mechanical fragility is fundamentally required before hydrogels can be reliably deployed in demanding environments. To improve the mechanical properties of PVA hydrogels, the freeze–thaw method has been widely adopted as a green and efficient physical crosslinking strategy [10,11,12]. This approach induces phase separation and crystallization through repeated freezing and thawing cycles, forming physically crosslinked networks without the need for additional chemical reagents. Compared with conventional chemical crosslinking methods, freeze–thaw processing avoids the use of potentially toxic crosslinkers and organic solvents, offering significant advantages in terms of safety, simplicity, and environmental friendliness. Previous studies have demonstrated that PVA hydrogels prepared via freeze–thaw cycling exhibit enhanced mechanical performance, with reported tensile strengths reaching approximately 2.5 MPa, confirming the effectiveness of this technique [13,14,15,16]. Although PVA hydrogels formed via freeze–thaw cycling exhibit improved mechanical properties compared with uncross linked systems, their performance in load-bearing applications remains suboptimal [17]. This limitation primarily arises from the relatively low crystallinity and insufficient physical crosslinking density generated during conventional freeze–thaw processes, which restrict effective stress transfer and structural integrity under high load or large deformation conditions. Moreover, excessive freeze–thaw cycles often lead to increased brittleness, further constraining the achievable balance between strength and toughness.

To address these mechanical challenges, the incorporation of the Hofmeister effect has recently emerged as a promising structural engineering strategy [3,17]. Specifically, kosmotropic ions (such as citrate and sulfate) strongly interact with water molecules, disrupting the polymer hydration shell and leading to salting-out. This drives enhanced intermolecular hydrogen bonding and severe network densification. While previous studies have shown that combining freeze–thawing with Hofmeister salting-out effectively reinforces the polymer network to achieve high tensile strength and toughness [18,19], the profound impact of such severe network densification and ion-polymer interactions on the thermal and combustion behaviors of hydrogels remains largely unexplored [20]. Currently, most modifications focus solely on load-bearing capabilities, failing to address the inherent vulnerability of hydrogels to extreme temperatures and fire [20,21,22]. While the Hofmeister effect radically reconstructs the micro-network, how such severe structural densification and specific ion-polymer interactions fundamentally affect the heat transfer and combustion behaviors of hydrogels remains completely unknown [23,24,25,26]. In practical high-end scenarios (e.g., flexible electronics and protective gear), materials require comprehensive thermal stability and flame retardancy alongside mechanical robustness [27,28,29,30]. Therefore, developing a strategy to integrate mechanical strength, thermal management, and flame retardancy into a single hydrogel architecture is highly desired but remains a critical challenge.

**Figure 1:**
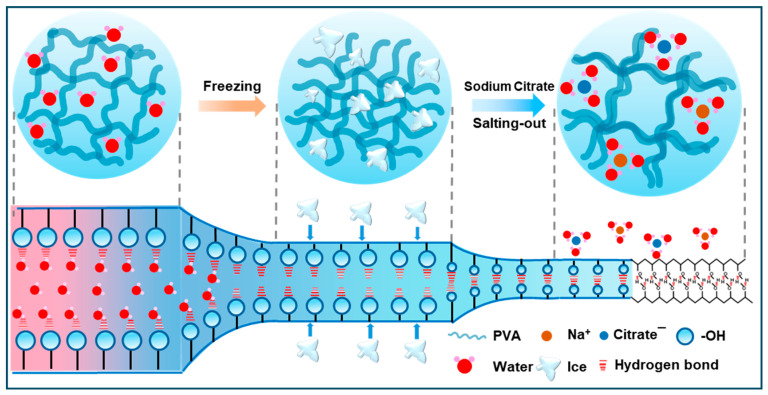
Preparation mechanism of PVA hydrogel.

Herein, we report a multifunctional PVA hydrogel that synergistically combines these three functionalities through a two-step strategy: freeze–thawing followed by citrate-driven Hofmeister salting-out. As illustrated in Figure 1, the initial freeze–thaw cycling constructs a primary physically crosslinked PVA skeleton. Subsequent immersion in a saturated sodium citrate solution induces a potent salting-out effect, which deeply densifies the polymer network while preserving its porous architecture. The resulting cit@PVA hydrogel exhibits balanced mechanical properties (tensile strength of 1.31 MPa, elongation at break of approximately 150%), high mid-infrared emissivity (~85%) enabling effective passive radiative cooling, and significantly enhanced flame retardancy through citrate-induced catalytic charring that forms a dense carbonaceous shield during combustion. This work demonstrates that the Hofmeister effect, beyond its well-recognized role in mechanical enhancement, can simultaneously regulate thermal and combustion behaviors, offering a scalable and integrated pathway toward resilient soft materials with combined mechanical robustness, thermal management, and fire safety.

## 2. Materials and Methods

### 2.1. Materials

Polyvinyl alcohol (PVA-1799) was purchased from Aladdin (Shanghai, China). Sodium citrate dihydrate, calcium chloride (CaCl_2_), and Sodium sulfate (Na_2_SO_4_) were supplied by Macklin (Shanghai, China). Deionized water was used in all aqueous solutions. All chemicals were used as received without further purification.

### 2.2. Fabrication of Hydrogels

PVA aqueous solutions with concentrations of 5, 6, 8, 10, and 12 wt% were prepared by dissolving the polymer in deionized water. The mixtures were heated to 95 °C under continuous stirring until homogeneous and transparent solutions were obtained. The resulting solutions were subsequently degassed by ultrasonication for 1 h to eliminate entrapped air bubbles. After degassing, the solutions were cast into silicone molds (with a thickness of 1.5 mm) and frozen at −20 °C for 24 h. Subsequently, without demolding, 20 mL of deionized water, saturated sodium citrate solution, saturated CaCl_2_ solution, or saturated Na_2_SO_4_ solution was directly poured into the molds onto the frozen samples. The system was then maintained at room temperature for 24 h. This in situ concurrent process allowed the samples to thaw and undergo ion-specific salting-out simultaneously, effectively preventing any potential mechanical damage to the delicate networks that might occur during premature demolding. Finally, the fully crosslinked hydrogels were carefully demolded and thoroughly washed with deionized water (200 mL per sample, renewed three times) to remove any residual unassociated ions from the hydrogel networks. After the washing step, the samples were gently wiped with filter paper to remove excess surface moisture prior to subsequent characterizations. No noticeable swelling of the hydrogel samples was observed during the whole fabrication process. For brevity, the PVA hydrogels treated with water, sodium citrate, calcium chloride, and sodium sulfate are denoted as water@PVA, cit@PVA, Ca@PVA, and SO_4_@PVA hydrogels, respectively.

### 2.3. Microstructural Observation

The morphology of the hydrogels was investigated using scanning electron microscopy (SEM, Hitachi SU8000, Tokyo, Japan). Prior to observation, hydrogel samples were freeze-dried at −38 °C for 24 h to preserve their internal microstructures. The dried samples were fractured to expose cross-sections and sputter-coated with a thin conductive gold layer (~10 nm) using a CCU-010 sputter coater (Sanyu Electron, Tokyo, Japan) before imaging to enhance electrical conductivity. SEM observations were carried out at different magnifications to analyze pore morphology, pore size distribution, and network uniformity.

### 2.4. Chemical Structure and Composition Analysis

To eliminate the interference of liquid water, all hydrogel samples used in this section were freeze-dried prior to characterization. The chemical structures and intermolecular interactions of the hydrogels were characterized by Fourier transform infrared spectroscopy (FTIR, Thermo Fisher iS55, Waltham, MA, USA) in the range of 4000–500 cm^−1^ at a resolution of 4 cm^−1^. Spectra were collected in ATR mode with 32 scans averaged for each sample to minimize noise. The spectra were used to identify characteristic functional groups and to evaluate changes in hydrogen bonding induced by salt treatment. The crystalline structure of the hydrogels was analyzed using X-ray diffraction (XRD, Rigaku Ultimate IV, Tokyo, Japan) equipped with Cu Kα radiation (λ = 1.5418 Å, weight average). Measurements were conducted at 40 kV and 45 mA with a scanning range of 5–80° and a scanning speed of 2°/min. XRD patterns were used to assess the effect of freeze–thaw processing and Hofmeister ion treatment on the crystallinity of PVA. Surface chemical composition and bonding environments were examined by X-ray photoelectron spectroscopy (XPS, Thermo Fisher 250Xi, Waltham, MA, USA). The full survey spectrum and high-resolution scans of C 1s and O 1s were acquired under ultra-high vacuum (residual pressure ~1 × 10^−6^ Pa) with a pass energy of 55.0 eV. Peak fitting was performed using XPS Peak 4.1 software to analyze changes in chemical states and intermolecular interactions.

### 2.5. Mechanical Property Evaluation

The mechanical properties of the as-prepared wet hydrogels were evaluated using a universal mechanical testing machine (Gotech, Dongguan, China) under tensile mode. The samples were cut into rectangular strips with dimensions of 35 mm × 10 mm × 1.5 mm (L × R × T). The samples were stretched at a constant strain rate of 20 mm/min until failure. Stress–strain curves were recorded to determine tensile strength, elongation at break, and toughness. Each sample was tested at least five times to ensure reproducibility.

### 2.6. Thermal Management and Thermal Stability Evaluation

Thermal management performance was evaluated using infrared thermal imaging on wet hydrogel samples (thickness: 1.5 mm). Hydrogel samples were placed on a temperature-controlled heating plate, which was heated from 30 °C to 180 °C at a constant rate over a total duration of 70 s. Surface temperature evolution was monitored using an infrared camera (FLIR Lepton, Wilsonville, OR, USA), and surface temperature distributions were recorded every 10 s to analyze heat transfer behavior. The solar reflectance was measured a UV–vis–NIR spectrophotometer (Thermo Fisher Nicolet iS50, Waltham, MA, USA). The obtained spectra were used to analyze the solar reflectance characteristics of the hydrogels and their potential contribution to radiative cooling performance. Thermal stability was assessed by thermogravimetric analysis (TGA, TA Instruments, New Castle, DE, USA). Approximately 10 mg of sample was heated from 30 °C to 600 °C at a heating rate of 10 °C/min under a nitrogen flow rate of 60 mL/min. The tests were repeated three times to ensure reliability, and weight loss as a function of temperature was recorded to evaluate thermal decomposition behavior and char residue formation. Derivative thermogravimetric (DTG) curves were obtained to analyze the maximum decomposition rate and the corresponding temperature.

### 2.7. Flame-Retardant Performance Evaluation

Flame-retardant performance was visually evaluated by a direct flame exposure test using the as-prepared wet hydrogels (thickness: 1.5 mm). The samples were vertically fixed and exposed to the jet flame of a commercial portable torch lighter (approximate temperature: ~1100 °C) at a fixed distance of 1.5 cm for a specific duration. The combustion process was recorded using a camera. Ignition behavior, flame propagation, and post-burning residue morphology were analyzed to assess the flame-retardant characteristics of the samples. Microscale combustion calorimetry (MCC) was performed using a microscale combustion calorimeter (FTT0001, Fire Testing Technology Ltd., West Sussex, UK). The samples were heated from room temperature to 750 °C at a rate of 1 °C/s.

## 3. Results and Discussion

### 3.1. Morphological Analysis

**Figure 2:**
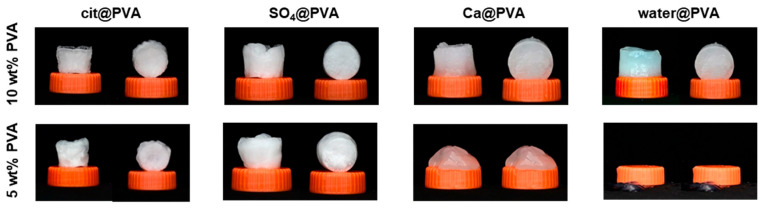
Macroscopic appearance of PVA hydrogels with different initial concentrations (5 wt% and 10 wt%) after various aqueous treatments.

The interplay between the Hofmeister effect and initial polymer concentration exerts a profound macroscopic influence on the gelation behavior and structural integrity of the PVA hydrogels. As illustrated in Figure 2, the macroscopic appearance of the hydrogels after immersion in different aqueous environments revealed significant differences. Among the investigated treatments, hydrogels treated with saturated kosmotropic salts, specifically cit@PVA and SO_4_@PVA, exhibited superior macroscopic robustness and a distinctly opaque appearance. This intense opacity is a macroscopic manifestation of severe polymer chain aggregation and crystallization triggered by the ion-induced salting-out effect. As a baseline reference, the water@PVA hydrogels maintained a translucent and relatively soft appearance. This indicates the inherent, looser physically crosslinked network formed solely by the initial freeze–thaw process, lacking the additional densification provided by specific ion-polymer interactions. Conversely, the Ca@PVA hydrogels demonstrated a pronounced salting-in effect, which severely disrupted the hydrogen bonding network and led to extreme gel swelling, high transparency, and eventual structural collapse. Furthermore, the macroscopic stability was highly dependent on the initial PVA concentration. Hydrogels prepared with 10 wt% PVA consistently demonstrated enhanced shape retention and structural support across all treatment groups. In contrast, reducing the concentration to 5 wt% significantly amplified the structural vulnerability of the networks. For instance, at 5 wt%, the water@PVA hydrogel displayed noticeable macroscopic sagging, while the Ca@PVA sample almost completely lost its structural integrity and collapsed onto the substrate. Despite these clear macroscopic trends, visual inspection alone was insufficient to distinguish the underlying microstructural evolution between the two highly densified systems (i.e., cit@PVA and SO_4_@PVA).

**Figure 3:**
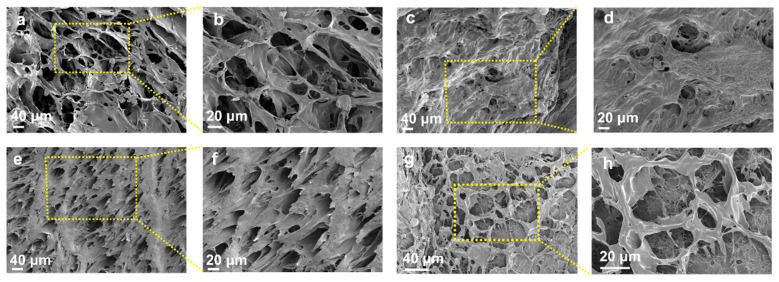
Cross-sectional SEM images of the hydrogels at different magnifications. (**a**,**b**) 5 wt% PVA treated with sodium citrate. (**c**,**d**) 10 wt% PVA treated with sodium citrate. (**e**,**f**) 5wt% PVA hydrogel treated with Na_2_SO_4_. (**g**,**h**) 10% PVA hydrogel treated with Na_2_SO_4_.

**Figure 4:**
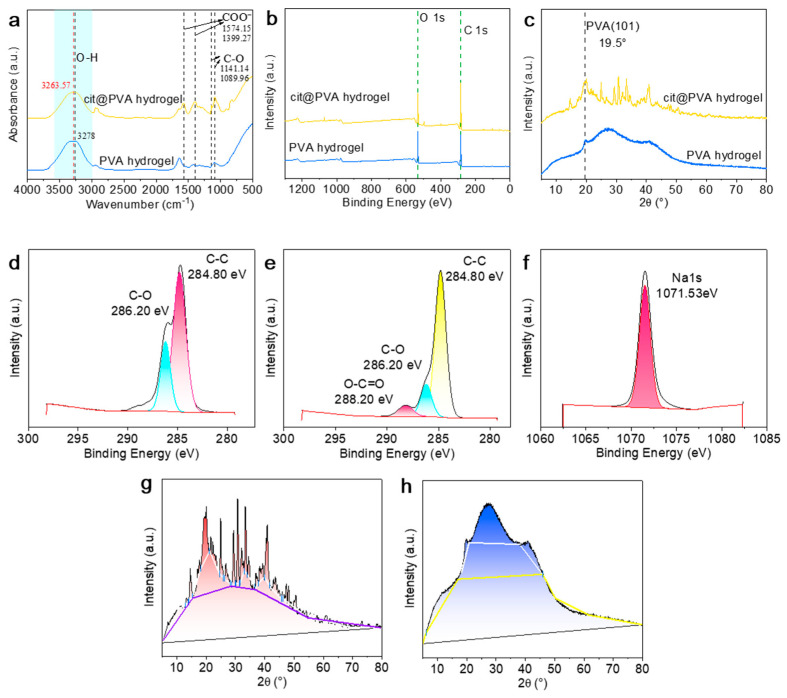
(**a**) FTIR spectra, (**b**) XRD patterns, and (**c**) XPS survey spectra of the water@PVA and cit@PVA hydrogel. High-resolution XPS C 1s spectra of the (**d**) water@PVA and (**e**) cit@PVA hydrogel. (**f**) High-resolution XPS Na 1s spectrum of cit@PVA hydrogel. (**g**) Schematic illustration of crystallinity calculation for cit@PVA. The black curve represents the original XRD pattern, and the white line represents the fitted crystalline diffraction peaks. The integrated area of crystalline diffraction peaks above the white line and the total diffracted area above the purple fitting curve were used to calculate the crystallinity. (**h**) Schematic illustration of crystallinity calculation for PVA using the same method, where the total diffracted area was obtained above the yellow fitting curve.

To further elucidate the structural origins underlying the macroscopic robustness, SEM was employed to visualize the internal micro-architectures of the cit@PVA and SO_4_@PVA hydrogels across different polymer concentrations (5 wt% and 10 wt%). As shown in Figure 3a–d, the cit@PVA hydrogels exhibit a well-defined, interconnected 3D porous network with continuous pore walls. Notably, increasing the initial PVA concentration from 5 wt% to 10 wt% refines the pore size and thickens the pore walls, yielding a densely packed yet structurally homogeneous architecture. In comparison, the SO_4_@PVA hydrogels display markedly different morphological features (Figure 3e–h). The 5 wt% SO_4_@PVA network exhibits directionally elongated and distorted pores (Figure 3e,f), a deformation that directly reflects its insufficient structural rigidity and looser crosslinking. Furthermore, the 10 wt% SO_4_@PVA (Figure 3g,h) exhibits a notably less densified porous architecture compared to the highly compacted cit@PVA network. This microstructural divergence stems from the distinct salting-out capacities of the kosmotropic ions. With its higher charge density and multiple carboxylate groups, the trivalent Cit^3−^ exerts a substantially stronger Hofmeister effect than the divalent SO_4_^2−^. It efficiently strips polymer hydration shells to drive extensive, templated hydrogen bonding, locking the PVA chains into an ultra-compact, rigid 3D skeleton that resists sectioning deformation. Notably, this ion-specific reinforcement cannot be attributed to a simple ionic-strength effect: saturated CaCl_2_, whose ionic strength is comparable to that of saturated Na_2_SO_4_, produced the opposite structural outcome—pronounced salting-in and swelling rather than densification, as described above for the Ca@PVA hydrogel—confirming that the observed reinforcement follows the specific ranking of the Hofmeister series rather than bulk ionic strength alone. This ion specificity, together with the markedly increased crystallinity (32.7% to 43.5%, Figure 4g,h) and the persistent citrate-related XPS signals detected even after extensive washing (Figure 4e,f), collectively indicates that citrate is specifically and stably incorporated into the hydrogel network through multidentate hydrogen-bond templating, rather than acting merely as a source of ionic strength. Quantitative image analysis was performed on the porous samples (PVA and SO_4_@PVA), while the cit@PVA hydrogel exhibited a highly densified morphology with indistinct pore boundaries, making conventional pore size analysis unsuitable. Therefore, the densification was evaluated qualitatively based on the disappearance of interconnected pores together with the reduced void area observed in SEM images. Consequently, the highly homogeneous and densely packed 10 wt% cit@PVA hydrogel was selected for subsequent investigations.

### 3.2. Chemical Structure and Intermolecular Interactions

To reveal the intrinsic molecular driving forces behind the observed microstructural densification, FTIR, XPS, and XRD were employed to systematically investigate the chemical compositions, intermolecular interactions, and crystallographic evolution of the hydrogels. As shown in Figure 4a, the water@PVA exhibits a broad absorption band centered at approximately 3278 cm^−1^, which corresponds to the stretching vibration of hydroxyl (O–H) groups. Upon treatment with sodium citrate, this O–H stretching peak undergoes a distinct red-shift to 3263 cm^−1^. In infrared spectroscopy, such a shift toward lower wavenumbers signifies a reduction in vibrational energy, unambiguously indicating the formation of a much denser and stronger intermolecular hydrogen-bonding network [31]. This phenomenon validates the potent salting-out effect of the kosmotropic citrate ions (Cit^3−^), which aggressively strip the hydration water shells surrounding the PVA chains, thereby forcing the exposed hydroxyl groups to heavily crosslink with each other. Moreover, two new characteristic peaks appear at approximately 1574 cm^−1^ and 1399 cm^−1^, which can be attributed to the asymmetric and symmetric stretching vibrations of carboxylate (COO^−^) groups originating from citrate ions [32,33]. The emergence of these peaks, alongside the slight shift in the C–O stretching region (from 1141 to 1090 cm^−1^), confirms that substantial intermolecular interactions are established between the PVA chains and the intercalated citrate ions.

Consequently, this intensified intermolecular hydrogen bonding directly governs the crystallographic transformation of the macroscopic material, as evidenced by the XRD patterns (Figure 4b). The water@PVA hydrogel displays a broad and relatively weak diffraction halo centered at approximately 19.5°, indicative of a typical semi-crystalline polymer with a loose structural order induced by simple physical freezing. Following the citrate salting-out treatment, the diffraction intensity of the (101) plane at 19.5° undergoes a dramatic enhancement, transforming from a broad hump into a highly sharp and intense crystalline peak. As shown in Figure 4g,h, Quantitative deconvolution of the XRD profiles was performed to estimate the crystallinity. Specifically, a baseline representing the total diffraction contribution was first constructed (yellow line for the water@PVA sample and purple line for the cit@PVA sample). The area enclosed between the experimental diffraction profile and the corresponding baseline was taken as the total diffraction area. Subsequently, the crystalline diffraction peaks were deconvoluted, and the integrated areas above the fitted amorphous envelope (white curves) were summed to obtain the crystalline peak area. The crystallinity was then calculated as the ratio of the crystalline peak area to the total diffraction area. Based on this method, the crystallinity increases markedly from approximately 32.7% for the water@PVA hydrogel to 43.5% after the citrate salting-out treatment, corresponding to a ~33.0% enhancement in the crystalline fraction. This remarkable increase in crystallinity confirms that the robust hydrogen bonds triggered by Cit^3−^ have successfully pulled the amorphous random coils of PVA into highly ordered, tightly packed crystalline domains. Furthermore, the multiple sharp diffraction peaks superimposed on the cit@PVA spectrum indicate the presence of confined sodium citrate microcrystals tightly entrapped within the highly condensed polymer pores during the drying process.

To further corroborate the chemical states and elemental composition of these densified networks and entrapped species, XPS analysis was performed. As illustrated in Figure 4c, both hydrogels exhibit prominent C 1s and O 1s signals. However, high-resolution deconvolution of the C 1s spectra reveals critical differences. For the water@PVA hydrogel (Figure 4d), the spectrum is deconvoluted into two classic components: the dominant peak at 284.80 eV corresponding to the aliphatic C–C/C–H backbone, and the peak at 286.20 eV assigned to the C–O bonds of the PVA hydroxyls. In stark contrast, the high-resolution C 1s spectrum of the cit@PVA hydrogel (Figure 4e) displays an additional, highly resolvable peak at 288.20 eV, which is the characteristic fingerprint of the O–C=O (COO^−^) groups from the citrate species. Concurrently, a strong Na 1s peak at 1071.53 eV emerges in the cit@PVA hydrogel (Figure 4f), which is completely absent in the pristine sample. These results comprehensively demonstrate that citrate ions are tightly anchored within the hydrogel matrix, altering its local chemical environment. Notably, even after extensive washing, the citrate-related XPS signals remained clearly detectable, further confirming the strong retention of citrate ions within the hydrogel network. Nevertheless, the present study did not quantitatively investigate the long-term leaching behavior of citrate ions. Further studies employing ion release analysis after prolonged immersion will be necessary to fully evaluate their long-term stability in practical applications.

In summary, the spectral and crystallographic evidence confirms that the synergistic combination of freeze–thaw and severe ion-induced salting-out yields an ultra-compact, highly crystalline, and densely hydrogen-bonded 3D network. Such a robust molecular architecture is anticipated to fundamentally dictate the macroscopic mechanical toughness and thermal stability of the hydrogels, which will be systematically evaluated in the subsequent sections.

### 3.3. Mechanical Properties

**Figure 5:**
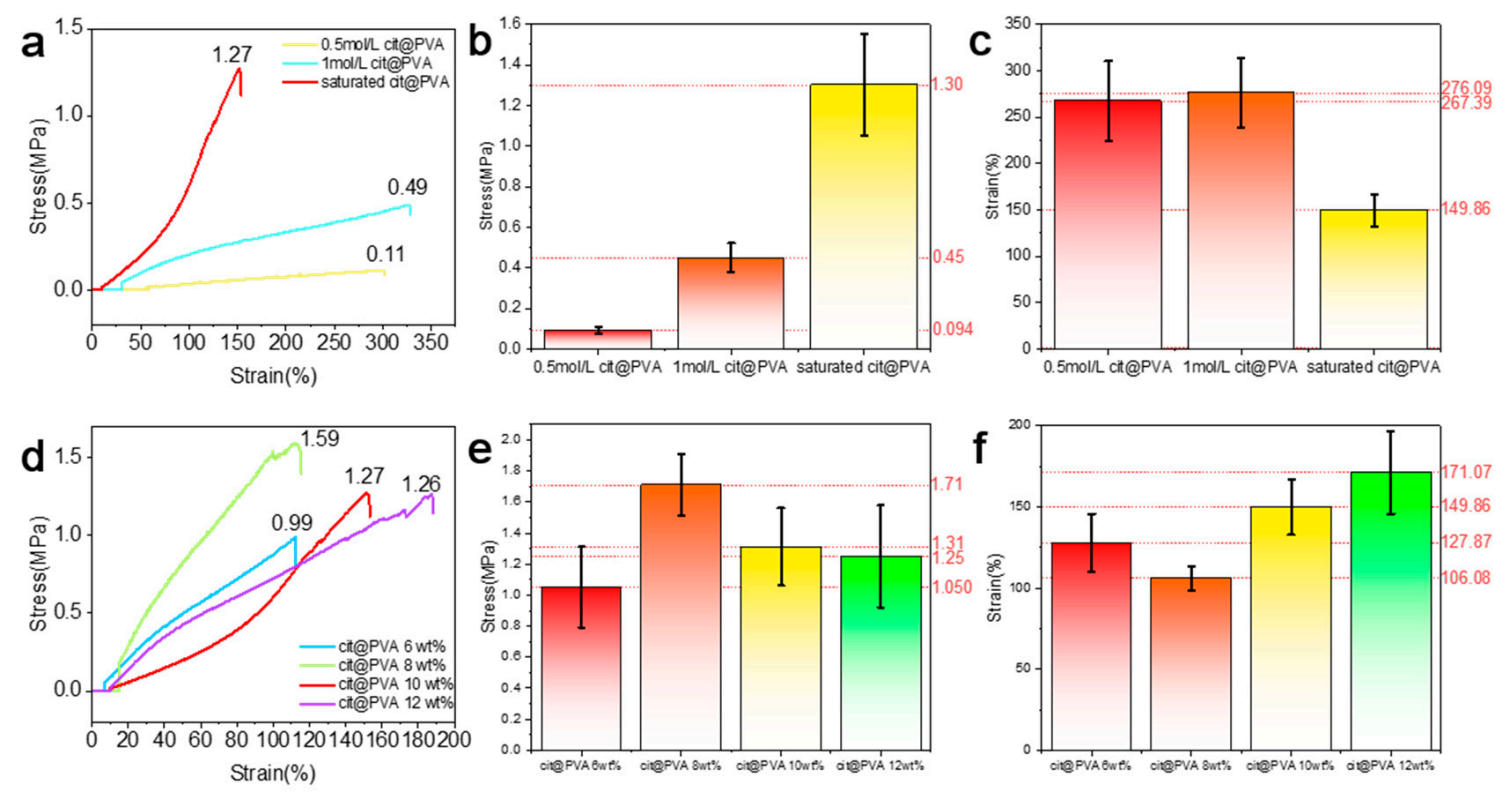
(**a**) Tensile stress–strain curves of the 10 wt% cit@PVA hydrogels treated with different sodium citrate concentrations (0.5 M, 1.0 M, and saturated). (**b**) Ultimate tensile stress and (**c**) elongation at break of the 10 wt% cit@PVA hydrogels treated with different sodium citrate concentrations. (**d**) Tensile stress–strain curves of the saturated sodium citrate-treated cit@PVA hydrogels prepared with different initial PVA concentrations (6, 8, 10, and 12 wt%). (**e**) Ultimate tensile stress and (**f**) elongation at break of the saturated sodium citrate-treated cit@PVA hydrogels prepared with different initial PVA concentrations.

To evaluate the mechanical performance and structural reliability of the cit@PVA hydrogels, tensile tests were systematically conducted. The effects of citrate concentration and initial PVA concentration on the mechanical properties were investigated, including tensile strength, elongation at break, and toughness (Figure 5).

The influence of citrate concentration on the mechanical reinforcement effect was first investigated using 10 wt% PVA hydrogels. As shown in Figure 5a,b, insufficient citrate concentration resulted in limited mechanical enhancement. The 0.25 mol/L citrate-treated sample was extremely soft and failed to provide reliable tensile measurements. With increasing citrate concentration, the tensile strength gradually increased from 0.094 ± 0.017 MPa for 0.5 mol/L cit@PVA to 0.45 ± 0.071 MPa for 1 mol/L cit@PVA. After treatment with saturated citrate solution, the tensile strength further increased to 1.31± 0.25 MPa, demonstrating the significant strengthening effect of concentrated citrate-induced salting-out. In contrast, the elongation at break followed a different trend with increasing citrate concentration (Figure 5c): it remained relatively high and comparable between 0.5 mol/L and 1 mol/L cit@PVA (267.39 ± 42.80% and 276.09 ± 37.40%, respectively), then decreased markedly to 149.86 ± 17.19% for the saturated citrate-treated sample, reflecting the restricted chain mobility associated with the highly densified network at high citrate concentration.

This concentration-dependent enhancement can be attributed to the progressive dehydration and densification of the PVA network induced by citrate ions. According to the Hofmeister effect, citrate ions possess strong hydration ability and can effectively reduce the water content surrounding PVA chains, thereby promoting closer polymer chain packing and enhancing intermolecular hydrogen-bond interactions. At relatively low citrate concentrations, only partial dehydration occurs, resulting in insufficient network contraction and limited mechanical reinforcement. In contrast, saturated citrate solution provides a stronger salting-out environment, facilitating more extensive dehydration and inducing a compact polymer network with improved stress-transfer capability.

The influence of initial PVA concentration on the tensile performance after saturated citrate treatment was further evaluated. As shown in Figure 5d,e, the tensile strength exhibited a non-monotonic dependence on PVA concentration. The average tensile strengths of 6, 8, 10, and 12 wt% cit@PVA hydrogels were 1.05 ± 0.26, 1.71 ± 0.20, 1.31 ± 0.25, and 1.25 ± 0.33 MPa, respectively. The 8 wt% sample exhibited the highest tensile strength, indicating that an appropriate polymer concentration facilitates the formation of an efficient load-bearing network. However, further increasing the PVA concentration did not continuously improve the tensile strength. The excessive polymer density may restrict the homogeneous rearrangement of polymer chains during citrate-induced dehydration, resulting in local structural heterogeneity and limiting further reinforcement.

In addition to tensile strength, the deformation capability of cit@PVA hydrogels was further analyzed through elongation at break and toughness evaluation. As shown in Figure 5d–f, the elongation behavior exhibited an opposite trend compared with tensile strength. The elongation at break increased from 106.08 ± 7.46% for 8 wt% cit@PVA to 171.07 ± 25.50% for 12 wt% cit@PVA. Meanwhile, the toughness values followed a similar variation trend, demonstrating that higher PVA concentrations contributed to enhanced energy dissipation capacity during deformation. This improvement can be attributed to the increased chain entanglement density, which enables more effective polymer chain sliding and rearrangement under external stress. In contrast, although the 8 wt% sample possessed the highest tensile strength, its relatively limited elongation indicated a more rigid network structure with reduced deformation tolerance.

Considering the balance between tensile strength, elongation, and mechanical stability, the optimal PVA concentration should not be determined solely based on the maximum tensile strength. Although the 8 wt% cit@PVA hydrogel exhibited the highest tensile strength (1.71 ± 0.20 MPa), its relatively low elongation at break indicated limited deformation tolerance. Increasing the PVA concentration to 12 wt% significantly improved the extensibility; however, the tensile strength did not show further enhancement and even slightly decreased to 1.25 ± 0.33 MPa, suggesting that the increased polymer content did not effectively contribute to further mechanical reinforcement. Moreover, the larger standard deviation observed for the 12 wt% sample indicates reduced mechanical consistency, which may be associated with increased difficulty in achieving a uniform network structure at higher polymer concentrations. In contrast, the 10 wt% cit@PVA hydrogel achieved a better balance among strength, deformability, and structural stability, exhibiting a relatively high tensile strength (1.31 ± 0.25 MPa), enhanced elongation (149.86 ± 17.19%), and lower mechanical variation. Therefore, the 10 wt% PVA concentration was selected as the optimized formulation for subsequent investigations.

### 3.4. Passive Radiative Cooling and Thermal Insulation

**Figure 6:**
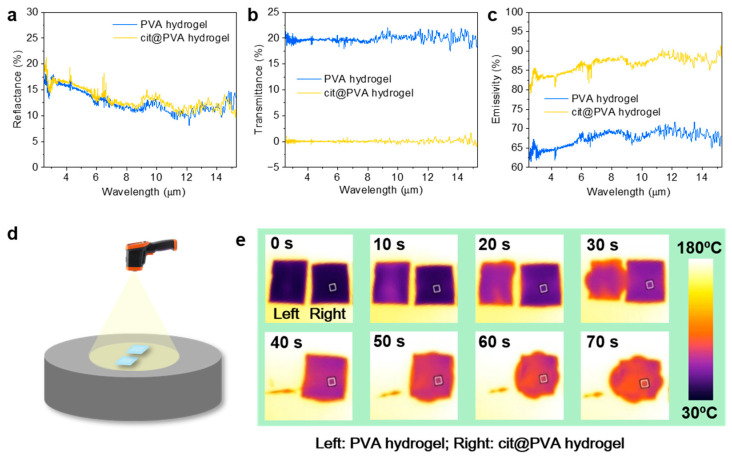
(**a**) Infrared reflection, (**b**) infrared transmission, and (**c**) infrared emissivity spectra of PVA hydrogel and cit@PVA hydrogel. (**d**) Schematic of the heating setup. (**e**) Time-dependent infrared thermal images monitoring the thermal evolution of the hydrogels on a hot plate.

To elucidate the thermal management capabilities arising from the highly densified micro-architecture, the intrinsic IR optical properties and macroscopic heat transfer behaviors of the hydrogels were systematically investigated. Initially, the fundamental IR optical characteristics were evaluated through spectral measurements. As shown in Figure 6a,b, the cit@PVA hydrogel exhibited a noticeably higher IR reflectance and a dramatic plummet in IR transmittance (approaching near-zero) compared with the water@PVA hydrogel. Notably, within the critical 3–8 μm wavelength range, the cit@PVA hydrogel demonstrates significantly elevated reflectance and minimized transmittance. This specific spectral feature is highly advantageous for passive radiative cooling, as it effectively shields the material from absorbing parasitic downward thermal radiation emitted by the ambient atmosphere. This stark optical transition from IR-transparent to IR-opaque is fundamentally governed by the intense Hofmeister salting-out effect. The densely packed micro/nanoporous networks and highly crystalline domains are considered to serve as effective Mie scattering centers, which strongly scatter and block the transmission of incident thermal radiation [34,35]. Concurrently, the cit@PVA hydrogel demonstrated a remarkably high and stable IR emissivity (Figure 6c), particularly across the primary atmospheric transparency window (8–13 μm). This enhancement is attributed to the synergistic combination of the densified intermolecular hydrogen-bonding network and the introduction of strongly vibrating carboxylate (COO^−^) functional groups. Consequently, by blocking environmental heat and maximizing thermal emission, this tailored spectral profile equips the material with superior passive radiative cooling to efficiently dissipate heat into space.

To evaluate the overall macroscopic thermal insulation capabilities of these engineered structures, a dynamic heating test was conducted. As illustrated in the schematic (Figure 6d) and the time-dependent infrared thermal images (Figure 6e), the hydrogels were placed directly onto a high-temperature hot plate. The water@PVA hydrogel exhibited a rapid and uncontrolled surface temperature rise, visually losing its dimensional stability as the heat swiftly penetrated the loose polymer network. In stark contrast, the cit@PVA hydrogel displayed a significantly delayed temperature response, maintaining a distinctly lower steady-state surface temperature and excellent shape retention even after 70 s of continuous exposure.

This exceptional thermal buffering performance is a direct macroscopic manifestation of its engineered structure. The near-zero IR transmittance effectively blocks direct radiative heat transfer from the hot substrate, while the highly tortuous porous skeleton and extensive crystalline domains severely disrupt phonon propagation, thereby minimizing solid thermal conductivity. Furthermore, the high surface emissivity synergistically pumps the accumulated heat into the cooler ambient environment. Regarding the thermal properties, the apparent contradiction between the thermal insulation observed in the present work and the thermal conductivity reported in other studies [25] stems from fundamentally different microstructural design principles. In Zhang et al. [25], mechanical stretching aligns PVA chains and forms oriented crystalline domains that act as efficient phonon transport pathways along the stretching direction. In contrast, our cit@PVA hydrogel achieves isotropic network densification via salting-out, creating a highly tortuous, randomly oriented porous skeleton with extensive crystalline domains. This isotropic architecture severely scatters and disrupts phonon propagation, minimizing solid thermal conduction. Simultaneously, the ultra-compact micro/nanoporous structure effectively scatters infrared radiation (Mie scattering), leading to high mid-infrared emissivity (~85%) that enables passive radiative cooling. Thus, by controlling the spatial organization of the densified network, the same Hofmeister-based densification strategy can be tuned to achieve either thermal insulation (isotropic densification) or, potentially, anisotropic thermal conduction if combined with directional processing.

### 3.5. Thermal Stability and Flame-Retardant Performance

**Figure 7:**
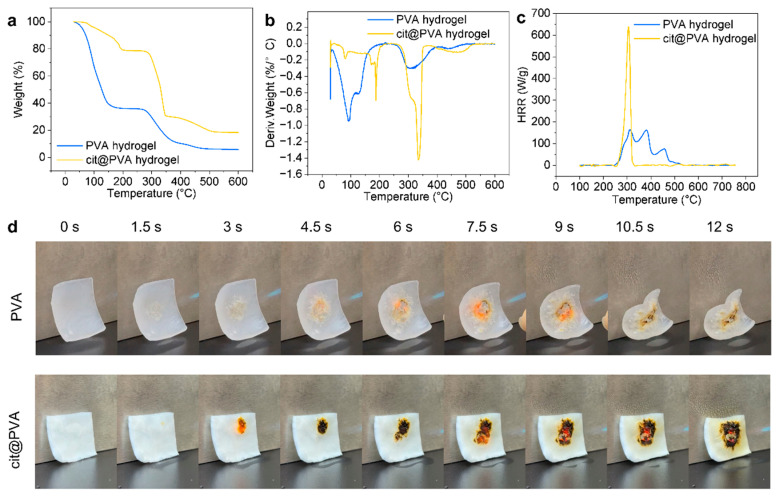
(**a**) Thermogravimetric analysis (TGA) curves, (**b**) derivative thermogravimetry (DTG) curves, and (**c**) heat release rate (HRR) of PVA hydrogel and cit@PVA hydrogel. (**d**) Time-lapse photographs showing the macroscopic combustion and charring processes of the PVA hydrogel and cit@PVA hydrogel under a direct flame.

To comprehensively evaluate the high-temperature survivability and fire safety of the densified networks under extreme thermal duress, the thermal stability and flame-retardant performance of the hydrogels were systematically investigated using the TGA, DTG, MCC, and direct flame exposure tests. The thermal degradation kinetics were initially elucidated through TGA and the corresponding DTG profiles. As shown in Figure 7a, PVA hydrogel exhibits a typical thermal degradation behavior characterized by a nearly complete mass loss at elevated temperatures, leaving a highly limited char residue. The DTG curve further corroborates this by displaying a broad and intense degradation peak associated with the complete structural collapse and bulk volatilization of the PVA backbone (Figure 7b). This indicates an unrestrained thermal decomposition dominated by the generation of combustible volatiles rather than carbonization. In stark contrast, the cit@PVA hydrogel demonstrates a fundamentally altered degradation pathway. While its DTG curve shows an earlier onset of initial mass loss, this early degradation step is precisely driven by the catalytic dehydration effect of sodium citrate. The citrate ions vigorously catalyze the cross-linking and carbonization of the PVA chains at lower temperatures. Consequently, the cit@PVA hydrogel successfully avoids catastrophic backbone fragmentation, ultimately yielding a significantly higher, thermally stable char residue at high temperatures.

The impact of this catalytic charring on the flammability of the pyrolysis products was further quantitatively assessed via the heat release rate (HRR) curves obtained from MCC (Figure 7c). The water@PVA hydrogel displays a broad HRR profile with a prolonged tail, indicating a continuous, unsuppressed evolution of combustible volatiles due to the lack of any protective physical barrier. In comparison, the cit@PVA hydrogel exhibits a markedly different profile, characterized by an earlier, sharper, and more concentrated HRR peak, followed by a rapid deceleration in heat release. Crucially, this apparent increase in the early-stage peak HRR does not imply aggravated flammability. Since MCC evaluates forced bulk pyrolysis and completely bypasses the macro-scale barrier effects of charring, this concentrated heat release is a direct reflection of the accelerated, citrate-catalyzed early surface carbonization. This rapid initial pyrolysis consumes the polymer surface to rapidly construct a protective char layer, which then effectively strangles further thermal decomposition at higher temperatures. The practical macro-scale protective efficacy of this unique “early-charring” mechanism is visually validated by the direct flame exposure tests (Figure 7d). During aggressive flame impingement, the water@PVA hydrogel rapidly softens, shrinks, and undergoes catastrophic structural collapse, burning vigorously until virtually completely destroyed. Conversely, the cit@PVA hydrogel exhibits exceptional structural integrity and pronounced flame resistance. Upon flame contact, the material avoids melt-dripping entirely; instead, the surface rapidly undergoes in situ carbonization. After the flame is removed, a continuous, compact, and robust black char layer remains, preserving the macroscopic shape of the hydrogel.

In summary, these thermal and combustion analyses demonstrate that the Hofmeister effect dictates a charring-dominated degradation pathway. The rapid formation of a dense carbonaceous shield effectively impedes heat transfer and volatile release, endowing the cit@PVA hydrogels with an exceptional synergy of thermal shielding and fire retardancy for extreme environments.

## 4. Conclusions

To overcome the inherent limitations of conventional PVA hydrogels, we engineered a multifunctional hydrogel integrating balanced mechanical properties, passive radiative cooling, and flame retardancy via synergistic freeze–thawing and citrate-driven Hofmeister salting-out. At the molecular and microstructural levels, kosmotropic citrate ions aggressively stripped polymer hydration shells, driving intense intermolecular hydrogen bonding, elevated crystallinity, and a deeply densified porous network. Consequently, the cit@PVA hydrogel achieved balanced mechanical performance with a tensile strength of 1.31 MPa and an elongation at break of approximately 150%. This compact architecture concurrently transformed its infrared optical properties, enabling high mid-infrared emissivity (~85%) for effective passive radiative cooling. Furthermore, the incorporated citrate shifted the degradation pathway toward catalytic charring, rapidly forming a dense carbonaceous shield during direct flame exposure to completely prevent burn-through. By synergistically integrating mechanical robustness, thermal management, and flame retardancy within a single physically crosslinked network, this scalable strategy yields resilient hydrogels with tremendous potential for flexible electronics, smart protective gear, and load-bearing devices in extreme environments.

## Data Availability

The original contributions presented in this study are included in the article. Further inquiries can be directed to the corresponding authors.
